# Incidence of RSV‐ and Influenza‐Associated Hospitalizations With Community‐Acquired Pneumonia and Other Acute Respiratory Infection Among Adults in Japan in 2022–2024: APSG‐J2 Study

**DOI:** 10.1111/irv.70238

**Published:** 2026-03-15

**Authors:** Haruka Maeda, Shingo Masuda, Bhim Gopal Dhoubhadel, Yuka Fujita, Yuji Akiba, Yutaka Nishigaki, Kei Nakashima, Hiroyuki Ito, Masayuki Nogi, Yoshihito Otsuka, Masayuki Ishida, Eiji Takeuchi, Norichika Asoh, Toyomitsu Sawai, Koichi Hayakawa, Eileen M. Dunne, Claudia Schwarz, Bradford D. Gessner, Elizabeth Begier, Shuhei Ito, Ataru Igarashi, Shinobu Osanai, Konosuke Morimoto, Koya Ariyoshi, Adult Pneumonia Study Group‐Japan 2 (APSG‐J2)

**Affiliations:** ^1^ Department of Respiratory Infections, Institute of Tropical Medicine Nagasaki University Nagasaki Japan; ^2^ Department of Clinical Medicine, Institute of Tropical Medicine Nagasaki University Nagasaki Japan; ^3^ Department of Respiratory Medicine National Hospital Organization Asahikawa Medical Center Asahikawa Japan; ^4^ Department of Respiratory Medicine Asahikawa‐Kosei General Hospital Asahikawa Japan; ^5^ Department of Pulmonology Kameda Medical Center Kamogawa Japan; ^6^ Department of General Internal Medicine Kameda Medical Center Kamogawa Japan; ^7^ Department of Clinical Laboratory Kameda Medical Center Kamogawa Japan; ^8^ Department of Infectious Disease Medicine, Department of Respiratory Medicine Chikamori Hospital Kochi Japan; ^9^ Department of Respiratory Medicine National Hospital Organization Kochi Hospital Kochi Japan; ^10^ Department of Internal Medicine Juzenkai Hospital Nagasaki Japan; ^11^ Department of Respiratory Medicine Nagasaki Harbor Medical Center Nagasaki Japan; ^12^ Department of Emergency Medicine Nagasaki Harbor Medical Center Nagasaki Japan; ^13^ Pfizer Vaccines Collegeville Pennsylvania USA; ^14^ Vaccine Medical Affairs, Pfizer Japan Inc. Tokyo Japan; ^15^ Department of Health Policy and Public Health Graduate School of Pharmaceutical Sciences, the University of Tokyo Tokyo Japan; ^16^ Division of Respiratory Medicine and Neurology, Department of Internal Medicine Asahikawa Medical University Asahikawa Japan

**Keywords:** adult, community‐acquired pneumonia, influenza, Japan, respiratory syncytial virus

## Abstract

**Background:**

Quantifying the burden of respiratory syncytial virus (RSV) in adults is challenging compared to influenza, and data among older adults remain scarce in Japan. Country‐specific evidence is essential to support RSV vaccination policy.

**Methods:**

This prospective, multicenter study (APSG‐J2) targeted hospitalized adults with community‐acquired pneumonia (CAP) and other acute respiratory infections (ARI) in seven community hospitals across four catchment areas in Japan between September 2022 and August 2024. Respiratory samples were analyzed using a multiplex polymerase chain reaction (PCR) kit to detect RSV and influenza. Incidence rates of RSV‐ and influenza‐associated hospitalizations were estimated using study data and national statistics, stratified by age and region.

**Results:**

Among 3047 hospitalized patients with CAP/ARI, 1499 (49.2%) underwent multiplex PCR testing. RSV and influenza were detected in 2.8% and 3.3% of tested patients, respectively. The incidences of RSV‐associated CAP/ARI hospitalizations among adults aged ≥ 65 years were 29 and 36 per 100,000 person‐years in the first and second years, respectively, with higher incidences among those aged ≥ 85 years (150 and 131 per 100,000 person‐years). Influenza incidence increased markedly in the second year (from 11 to 71 per 100,000 person‐years for adults age ≥ 65 years), possibly reflecting post‐COVID‐19 transmission changes.

**Conclusions:**

In this multicenter study, we estimated the incidence of RSV‐ and influenza‐associated hospitalizations among adults in Japan. The findings indicated that the incidence increased with age, and influenza‐associated hospitalizations increased in the second year. Continued surveillance is essential to accurately assess RSV burden in the adult population.

## 1 Introduction

Acute respiratory infections (ARIs) are a leading cause of morbidity and mortality, particularly among older adults [1]. Respiratory syncytial virus (RSV) is a well‐recognized viral pathogen primarily known for affecting infants [2, 3]. Recent evidence suggests a significant burden among older adults [4, 5, 6]. However, quantifying the true burden of RSV in adults is challenging compared to influenza, as RSV testing is not routinely performed in clinical practice, and testing sensitivity in adults is lower compared to infants due to the reduced viral load and other factors [7, 8]. Much of the previous evidence has relied on modeling methods using time‐series analysis [9, 10, 11, 12, 13], and retrospective studies based on clinical diagnoses [14, 15, 16]. Ideal studies require active surveillance using laboratory diagnostic tests to avoid underestimating the true burden of RSV infection [17, 18, 19, 20].

To date, in Japan, two kinds of RSV vaccines were approved for use in adults aged ≥ 60 years [21]. Neither of the RSV vaccines for older adults has yet been incorporated into Japan's National Immunization Program (NIP), while influenza vaccines for this age group have been included in the NIP since 2001, with coverage of approximately 50%–60% between 2020 and 2023 [22]. Because both RSV and influenza are vaccine‐preventable respiratory infections, understanding their disease burden is essential to inform vaccine policy decisions. In particular, country‐specific and age‐specific baseline data on disease burden prior to RSV vaccine implementation are crucial for evaluating the potential impact of vaccine introduction. However, data on burden of RSV among older adults in Japan remain scarce, especially regarding RSV‐associated hospitalizations [23, 24, 25, 26].

Our research team previously conducted the Adult Pneumonia Study Group–Japan (APSG‐J) surveillance between 2012 and 2014 at four hospitals located on each of Japan's major islands [27]. This multicenter study contributed critical insights into the epidemiology of community‐acquired pneumonia (CAP) in adults. During this study period, RSV was detected in 4.3% and influenza A/B in 4.7% among 2037 adults aged ≥ 65 years with CAP [28]. Since that initial surveillance, multiple changes, including the COVID‐19 pandemic, have influenced the epidemiological landscape of respiratory infections in Japan. In light of these changes, we initiated the second phase of the APSG‐J surveillance (APSG‐J2), aiming to reassess the incidence and etiological spectrum of CAP and other ARI.

In this study, we aimed to estimate the incidence of RSV‐ and influenza‐associated CAP and other ARI hospitalizations based on the APSG‐J 2 surveillance conducted between September 1, 2022, and August 31, 2024. We also assessed the clinical characteristics and outcomes of affected patients.

## 2 Methods

### 2.1 APSG‐J2 Surveillance

The APSG‐J2 is an ongoing, prospective, multicenter, hospital‐based study launched in September 2022 in adults in Japan. It is being conducted in seven community hospitals located in four catchment areas, known as “Niji‐Iryouken” (secondary medical areas): Asahikawa, Kamogawa, Kochi, and Nagasaki. Secondary medical areas (“Niji‐Iryoken”) are administratively designated units defined by prefectural governments under the Japanese Medical Care Act (a national law) (Supplementary Section 1). Although all seven hospitals are community‐based, the hospital in Kamogawa also functions as a tertiary referral center in the region and therefore applies stricter admission criteria, focusing on more severe patients. These areas, selected from Japan's four main islands, have populations ranging from approximately 30,000 to 400,000, except Kamogawa, with a population of < 30,000. The study targets hospitalized adults aged ≥ 18 years diagnosed with CAP or other ARI who resided in each catchment area (secondary medical area). Detailed inclusion and exclusion criteria are provided in Supplementary Section 1.

Supplementary Figure S1 outlines the screening and enrollment process conducted by study staff and clinicians at each site. For patients with whom consent could not be coordinated, enrollment followed an “opt‐out” approach in line with Japanese research ethics guidelines. For individuals who declined participation after being informed, minimal data (age, sex, and diagnosis) were collected to support incidence estimation. Demographic and clinical data were collected from electronic medical records and interviews with participants or proxies and recorded in an electronic case report form using REDCap [29].

This study includes patients enrolled between September 1, 2022, and August 31, 2024, a period that partially overlapped with strict public health measures implemented under Japan's Infection Control Law in response to the COVID‐19 pandemic. These measures remained in place until May 8, 2023, when the government reclassified COVID‐19 as a Category V infectious disease, equivalent to seasonal influenza.

### 2.2 Multiplex PCR for Respiratory Bacteria and Virus

For patients who provided informed consent for specimen collection, respiratory samples, such as sputum, saliva, or nasopharyngeal swabs, were collected and analyzed using a commercially available multiplex polymerase chain reaction (PCR) respiratory panel (Fast‐Track Diagnostics Respiratory Pathogen 33 assay, Siemens Healthineers, Germany) at a commercial laboratory (LSI Medience, Japan). This test detects the following 33 kinds of respiratory pathogens: influenza A, influenza A subtype A(H1N1)pdm09, influenza B, and influenza C; parainfluenza viruses 1, 2, 3, and 4; coronaviruses NL63, 229E, OC43, and HKU1; human metapneumoviruses A and B; rhinovirus; RSV A and B; adenovirus; enterovirus; parechovirus; bocavirus; *Pneumocystis jirovecii*; *Mycoplasma pneumoniae* ; *Chlamydia pneumoniae* ; *Streptococcus pneumoniae* ; *Haemophilus influenzae* ; *Haemophilus influenzae* type B; *Staphylococcus aureus* ; *Moraxella catarrhalis* ; *Bordetella species* (excluding *Bordetella parapertussis* ); *Klebsiella pneumoniae* ; *Legionella* species; and *Salmonella* species.

### 2.3 Definitions

A case of CAP was defined as an ARI with new pulmonary infiltrates on chest x‐ray or CT consistent with pneumonia, including increased pulmonary density, alveolar infiltrates with air bronchograms, or pleural effusion. Patients without present radiological evidence of CAP were categorized as other ARI. Hospitalized cases of CAP and other ARIs were considered RSV‐ or influenza‐positive if any collected specimens tested positive for RSV, influenza A, or B by the multiplex PCR, irrespective of co‐detection of other respiratory pathogens.

### 2.4 Statistical Analysis

Continuous variables are presented as medians with interquartile ranges (IQRs), while categorical variables are presented as counts and proportions. Demographics and characteristics of hospitalized patients with CAP and other ARIs who underwent multiplex PCR and those tested positive for RSV or influenza are described.

**FIGURE 1:**
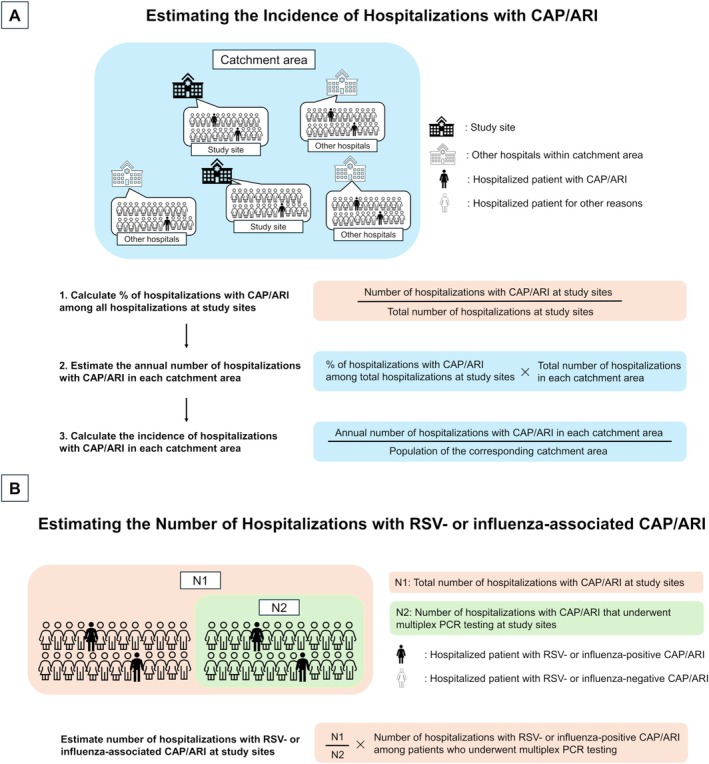
(A) Overview of the steps to estimate the incidence of hospitalizations with CAP and other ARI in each catchment area. First, the percentage of hospitalizations with CAP and other ARI at study sites within each catchment area was calculated by dividing the number of hospitalizations with CAP and other ARI at study sites by total number of hospitalizations at the corresponding sites. This percentage was then applied to the entire catchment area by multiplying it by the total number of hospitalizations in each catchment area to estimate the annual number of hospitalizations with CAP and other ARI in each catchment area. Finally, the incidence of hospitalizations with CAP and other ARI in each catchment area was calculated by dividing the estimated annual number of hospitalizations with CAP and other ARI in each catchment area by the population of the corresponding catchment area. (B) Overview of estimating the number of hospitalizations with RSV‐ or influenza‐associated CAP and other ARI at study sites. ARI, acute respiratory infection; CAP, community‐acquired pneumonia.

Incidence rates were calculated separately for two enrollment periods: September 2022–August 2023 and September 2023–August 2024. To estimate the incidence of hospitalized CAP and other ARI as well as those associated with RSV and influenza, we utilized study data alongside publicly available national statistics, applying a modified model used in a previous study [27]. The total number of CAP and other ARI hospitalizations, both overall, by study sites at each catchment area, and by study year, was estimated by combining data from three arms (Supplementary Figure S1): (1) patients who consented, (2) patients included in the surveillance arm under opt‐out consent, and (3) patients who declined but had minimal data collection. Age‐specific total number of CAP and other ARI hospitalizations for each study year was obtained by summing the numbers across all study sites within each catchment area (Figure 1A). We then calculated the proportion of hospitalized CAP and other ARI among all hospitalizations for each year at study sites in each catchment area by dividing the number of CAP and other ARI hospitalizations by the total number of hospitalizations at the same study sites in each catchment area during the corresponding year. To account for patients residing outside the designated catchment area, we adjusted the denominator by multiplying the total number of hospitalizations by one minus the proportion of patients from outside the catchment area [30]. For all catchment areas except Kamogawa, which had only one participating hospital, data from two hospitals were aggregated to calculate these proportions. These proportions were then multiplied by the total number of hospitalizations in each respective catchment area, based on government statistics [30], to estimate the annual total number of CAP and other ARI hospitalizations. Detailed estimation procedures are provided in Supplementary Section 1. Incidence rates were calculated by dividing the estimated annual number of CAP and other ARI hospitalizations in each catchment area by the population of the corresponding catchment areas, based on 2020 national census data [31, 32]. Annual incidence rates are expressed per 100,000 population. The 95% confidence intervals (CIs) for the proportion of CAP and other ARI hospitalizations among all hospitalized patients in each catchment area were first calculated using the Wald method (*p* ± 1.96 × standard error), and these intervals were then propagated to derive 95% CIs for the estimated annual number of CAP and other ARI hospitalizations and the corresponding incidence rates. These estimates were stratified by age group to provide age‐specific incidence. Population data for catchment areas were provided in 5‐year age intervals. As only data for individuals aged ≥ 25 years were available, incidence estimates were restricted to this population. Incidence was reported for the following age groups: ≥ 25, ≥ 65, ≥ 75, 65–74, 75–84, and ≥ 85 years.

To estimate incidence rates of RSV‐ or influenza‐associated CAP and other ARI hospitalizations, the same method described above was applied, except that the total number of RSV‐ or influenza‐associated hospitalizations was used in place of the total number of CAP and other ARI hospitalizations. To calculate the number of RSV‐ or influenza‐associated CAP and other ARI hospitalizations by age group for each study year by study sites in each catchment area, we multiplied the number of RSV‐ or influenza‐positive CAP and other ARI hospitalizations among those who underwent multiplex PCR by the ratio of the total number of CAP and other ARI hospitalizations to the number of such hospitalizations with multiplex PCR (Figure 1B). This approach assumes that patients who were not tested had similar characteristics (and therefore a similar proportion of RSV or influenza positivity) to those who underwent testing.

As a sensitivity analysis, we recalculated the overall incidence rates of RSV‐ or influenza‐associated CAP and other ARI hospitalizations among adults aged ≥ 65 years after excluding Kamogawa, where the hospital functions as a large referral center and may differ from the other hospitals in characteristics such as admission thresholds and referral patterns.

To examine the effect of age, we compared incidence rates of total, RSV‐associated, and influenza‐associated hospitalizations between younger adults (25–64 years) and older adults (≥ 65 years) by calculating the incidence rate ratios (IRRs) and 95% CIs using the log‐normal approximation [33]. The younger age group (25–64 years) was used as the reference category. A two‐sided *p* value of < 0.05 was considered statistically significant.

All analyses were conducted using Stata version 17.0 (StataCorp, College Station, TX, USA).

### 2.5 Ethics

This study was approved by the institutional review board of the Institute of Tropical Medicine, Nagasaki University with a centralized review (Approval No.: 220616247).

## 3 Results

**FIGURE 2:**
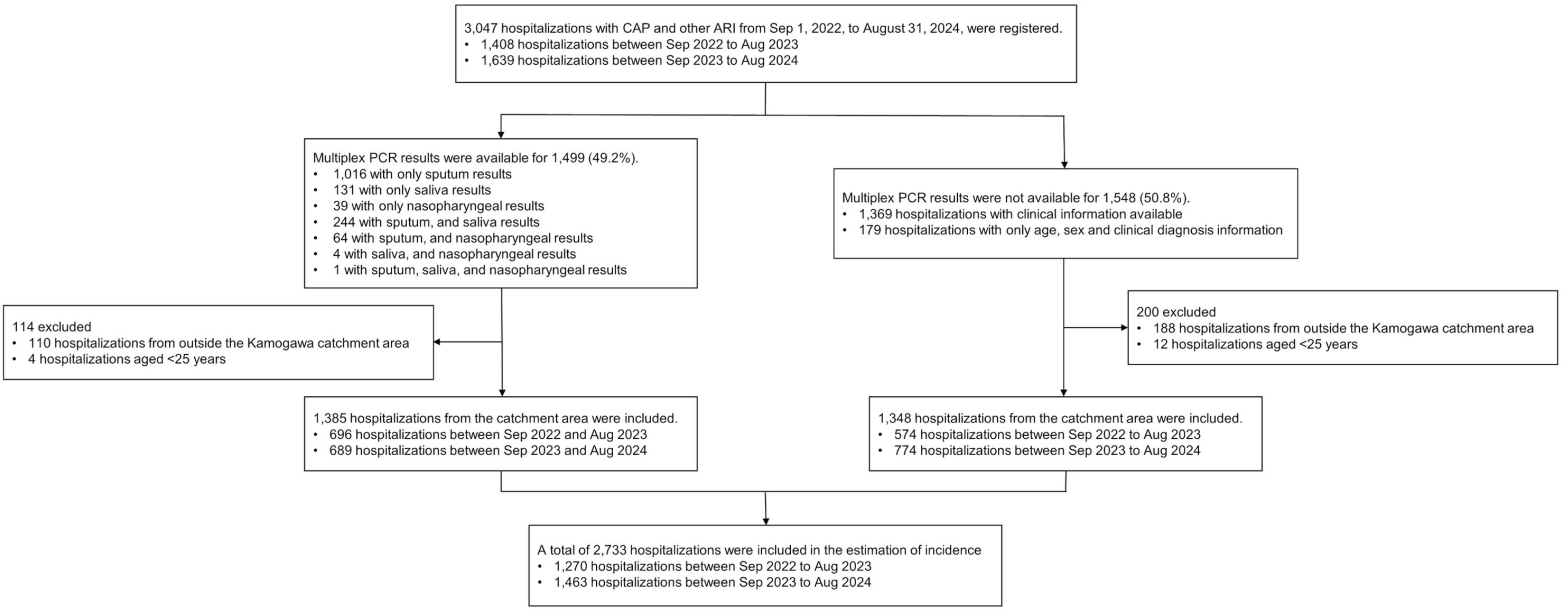
Flowchart of patient inclusion for CAP and other ARI from September 1, 2022, to August 31, 2024, in Japan. A total of 3047 hospitalizations were registered, of which multiplex PCR results were available for 1499 (49.2%) hospitalizations. Among all registered hospitalizations, 2733 hospitalizations were in the analysis for incidence estimation. ARI, acute respiratory infection; CAP, community‐acquired pneumonia.

A total of 3047 hospitalizations with CAP and other ARI were identified across four catchment areas between September 1, 2022, and August 31, 2024: 1408 in the first year and 1639 in the second year. Multiplex PCR results were available for 1499 patients (49.2%) (Figure 2). The characteristics of patients who underwent testing were generally similar to those who did not (Supplementary Section 2, Supplementary Table S1). Among the 1499 tested patients, sputum, saliva, and nasopharyngeal swab were collected from 1325 (88.4%), 380 (25.4%), and 108 (7.2%) patients, respectively (Supplementary Table S2).

### 3.1 Characteristics of Patients Hospitalized With CAP and Other ARI Who Underwent Multiplex PCR

Table 1 shows the characteristics of 1499 patients hospitalized with CAP and other ARI who underwent multiplex PCR. The median age was 81 years (IQR: 73–88), and 64.1% were male. Overall, 82.3% had at least one underlying condition, 17.5% were nursing home residents, and 36.6% required some form of care (Supplementary Table S5). The most common underlying conditions were chronic lung disease (40.3%), chronic heart disease (33.8%), and diabetes mellitus (23.2%). Patient distribution was similar across the 2 years. Clinically, 93.5% of patients underwent chest CT at admission, 7.9% were admitted to ICU, 6.5% required mechanical ventilation, and in‐hospital mortality was 8.0%. The median length of hospitalization was 15 days (IQR: 10–27), and 90.7% were diagnosed with CAP (Table 1).

**TABLE 1:** Characteristics of adults hospitalized with CAP and other ARI who underwent multiplex PCR, with RSV‐ and influenza‐positive CAP and other ARI in Japan, September 2022–August 2024.

	Overall, no. (%) (*n* = 1499)	RSV‐positive, no. (%) (*n* = 42)	Influenza‐positive, no. (%) (*n* = 49)
Median age (IQR), years	81 (73–88)	82 (76–92)	79 (71–88)
Age group in years			
18–64	184 (12.3)	5 (11.9)	7 (14.3)
≥65	1315 (87.7)	37 (88.1)	42 (85.7)
65–74	262 (17.5)	4 (9.5)	10 (20.4)
75–84	489 (32.6)	14 (33.3)	13 (26.5)
≥85	564 (37.6)	19 (45.2)	19 (38.8)
Sex			
Men	961 (64.1)	19 (45.2)	33 (67.3)
Women	538 (35.9)	23 (54.8)	16 (32.7)
Underlying medical conditions^a^			
Any	1233 (82.3)	33 (78.6)	41 (83.7)
Asplenia	1 (0.1)	0	0
Cancer	234 (15.6)	7 (16.7)	7 (14.3)
Leukemia	2 (0.1)	1 (2.4)	0
Lymphoma	10 (0.7)	0	1 (2.0)
Multiple myeloma	1 (0.1)	0	0
Immunosuppressive drug use	73 (4.9)	4 (9.5)	4 (8.2)
Organ transplantation	2 (0.1)	0	0
Diabetes mellitus	348 (23.2)	11 (26.2)	10 (20.4)
Nephrotic syndrome	3 (0.2)	0	0
Chronic heart failure	264 (17.6)	6 (14.3)	7 (14.3)
Chronic heart disease other than chronic heart failure	343 (22.9)	9 (21.4)	16 (32.7)
Chronic lung disease (including asthma)	604 (40.3)	16 (38.1)	28 (57.1)
Chronic renal failure	146 (9.7)	3 (7.1)	6 (12.2)
Chronic live disease	68 (4.5)	3 (7.1)	2 (4.1)
Obesity (BMI ≥ 40)	4 (0.3)	0	0
Cerebrospinal fluid leakage	1 (0.1)	0	0
Cerebrovascular disease	270 (18.0)	7 (16.7)	5 (10.2)
Current smokers			
Yes	134 (8.9)	2 (4.8)	5 (10.2)
No	1284 (85.7)	38 (90.5)	43 (87.8)
Unknown	81 (5.4)	2 (4.8)	1 (2.0)
Alcohol dependency			
Yes	11 (0.7)	1 (2.4)	0
No	1453 (96.9)	41 (97.6)	49 (100)
Unknown	35 (2.3)	8 (19.0)	6 (12.2)
Nursing‐home residents	262 (17.5)	8 (19.0)	6 (12.2)
Nursing‐care level^b^			
Independent	804 (53.6)	27 (64.3)	27 (55.1)
Support 1	80 (5.3)	1 (2.4)	4 (8.2)
Support 2	65 (4.3)	1 (2.4)	6 (12.2)
Care level 1	135 (9.0)	3 (7.1)	7 (14.3)
Care level 2	106 (7.1)	1 (2.4)	2 (4.1)
Care level 3	108 (7.2)	4 (9.5)	1 (2.0)
Care level 4	117 (7.8)	3 (7.1)	1 (2.0)
Care level 5	82 (5.5)	2 (4.8)	1 (2.0)
Unknown	2 (0.1)	0	0
Prior hospitalization within the past 90 days			
Yes	290 (19.3)	6 (14.3)	6 (12.2)
No	1185 (79.1)	36 (85.7)	43 (87.8)
Unknown	24 (1.6)	0	0
Preceding antibiotics use within 14 days			
Yes	298 (19.9)	10 (23.8)	4 (8.2)
No	1188 (79.3)	32 (76.2)	45 (91.8)
Unknown	13 (0.9)	0	0
Influenza vaccination (most recent season)^c^			
Yes	624 (41.6)	20 (47.6)	20 (40.8)
No	503 (33.6)	12 (28.6)	27 (55.1)
Unknown	372 (24.8)	10 (23.8)	2 (4.1)
Study period			
First year (September 2022–August 2023)	754 (50.3)	17 (40.5)	6 (12.2)
Second year (September 2023–August 2024)	745 (49.7)	25 (59.5)	43 (87.8)
Area			
Asahikawa	218 (14.5)	5 (11.9)	6 (12.2)
Kamogawa	198 (13.2)	7 (16.7)	3 (6.1)
Kochi	780 (52.0)	21 (50.0)	29 (59.2)
Nagasaki	303 (20.2)	9 (21.4)	11 (22.4)
Diagnosis			
Community‐acquired pneumonia	1359 (90.7)	36 (85.7)	35 (71.4)
Other acute respiratory infection	140 (9.3)	6 (14.3)	14 (28.6)
Respiratory failure^d^ at the time of admission			
Yes	848 (56.6)	26 (61.9)	26 (53.1)
No	627 (41.8)	15 (35.7)	23 (46.9)
Unknown	24 (1.6)	1 (2.4)	0
Clinical outcomes			
ICU admission (*n* = 1497^e^)	119 (7.9)	2 (4.8)	0
Mechanical ventilation (*n* = 1497^e^)	98 (6.5)	2 (4.8)	2 (4.1)
Median duration of hospitalization (IQR), days	15 (10–27)	13 (9–22)	10 (6–17)
In‐hospital death	120 (8.0)	3 (7.1)	1 (2.0)

^a^ No patients had sickle cell disease, HIV infection, Hodgkin's disease, or cochlear implant.

^b^ Nursing care level was determined based on the Long‐Term Care Insurance System by the Ministry of Health, Labour and Welfare. Details are provided in Supplementary Table S5.

^c^ History of influenza vaccination in the most recent season (October–December) before the survey date.

^d^ Respiratory failure was defined as SpO_2_ < 90% or the need for oxygen administration.

^e^ Information on ICU admission and use of mechanical ventilation for the two hospitalizations is currently unavailable, as follow‐up is still in progress.

Abbreviations: ARI, acute respiratory infection; BMI, body mass index; CAP, community‐acquired pneumonia; ICU, intensive care unit; IQR, interquartile range; PCR, polymerase chain reaction.

### 3.2 Patients Hospitalized With CAP and Other ARI Positive for RSV and Influenza

**FIGURE 3:**
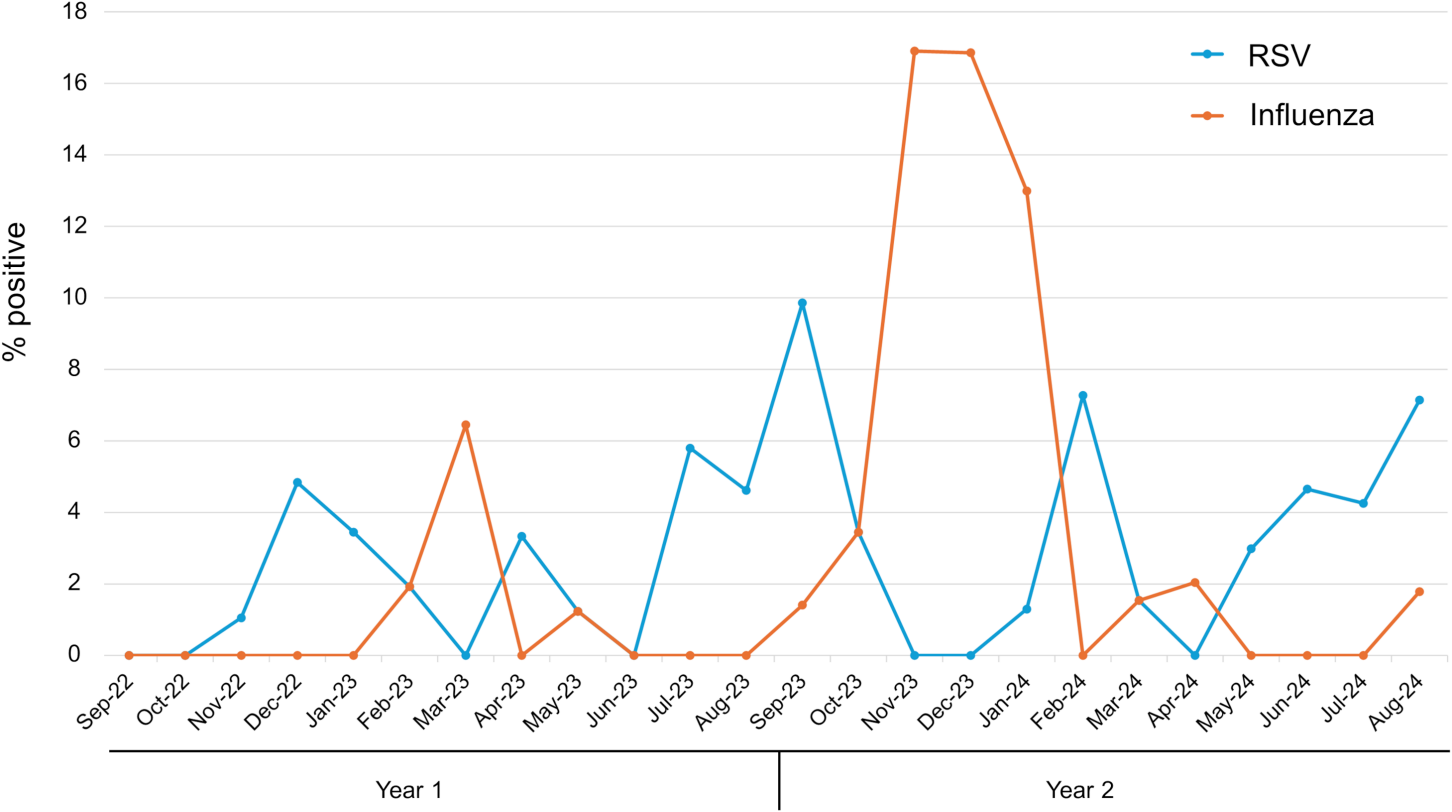
Monthly percentage of RSV‐ and influenza‐positive hospitalizations among community‐acquired pneumonia and other acute respiratory patients who underwent multiplex PCR from September 2022 to August 2024 in Japan. The blue line represents the percentage of RSV‐positive hospitalizations, while the orange line represents influenza‐positive hospitalizations. RSV, respiratory syncytial virus.

Figure 3 shows the monthly percentage of RSV‐ and influenza‐positive hospitalizations among CAP and other ARI patients who underwent multiplex PCR. Overall, 42 of 1499 patients (2.8%) tested positive for RSV: 2.3% and 3.4% in the first and second year, respectively. Influenza was detected in 49 patients (3.3%): 0.8% and 5.8% in the first and second year, respectively (Supplementary Table S3). One patient tested positive for both RSV and influenza. In addition, one RSV‐positive patient was also positive for parainfluenza 3, and one influenza‐positive patient was also positive for coronavirus OC43.

Two distinct peaks in influenza‐positive hospitalizations were observed: a larger peak during the 2023–2024 winter and a smaller peak in March 2023. In contrast, RSV‐positive hospitalizations exhibited multiple, less distinct peaks without a clear seasonal pattern with a 10% proportion of positive cases in the highest month.

Table 1 and Supplementary Table S4 summarize characteristics and symptoms of RSV‐ and influenza‐positive hospitalizations. Compared with influenza‐positive patients, those with RSV were older, more likely to be female, and more frequently residents of nursing home. CAP diagnoses were more frequent among RSV‐positive patients. Clinically, 61.9% (26/42) of RSV‐positive hospitalizations and 53.1% (26/49) of influenza‐positive hospitalizations experienced respiratory failure, defined as SpO_2_ < 90% or the need for oxygen at the time of admission. ICU admission was required in two RSV‐positive patients (4.8%), whereas none among influenza‐positive patients. Mechanical ventilation was administered to two patients in each group (4.8% for RSV, 4.1% for influenza). In‐hospital deaths occurred in three RSV‐positive (7.1%) and one influenza‐positive patient (2.0%).

### 3.3 Incidence of Total, RSV‐Associated, and Influenza‐Associated CAP and Other ARI

Table 2 presents the incidence rates of hospitalizations for total CAP and other ARI, as well as those associated with RSV or influenza. Among adults aged ≥ 25 years, the incidence of total CAP and other ARI hospitalizations per 100,000 person‐years was 483 (95% CI: 456–509) and 539 (95% CI: 511–566) per 100,000 person‐years in the first and second year, respectively. Among adults aged ≥ 65 years, corresponding incidence rates were 1113 (95% CI: 1050–1176) and 1217 (95% CI: 1152–1281) per 100,000 person‐years, respectively.

**TABLE 2:** Incidence rates of adults hospitalized with total, RSV‐associated, and influenza‐associated CAP and other ARI in Japan, September 2022–August 2024.

Hospitalized CAP and other ARI incidence rate per 100,000 person‐years (95% CI)					
Age group (years)	Overall	Asahikawa	Kamogawa	Kochi	Nagasaki
**Year 1: September 2022 to August 2023**					
≥ 25	483 (456–509)	516 (461–571)	123 (101–144)	689 (631–748)	666 (592–739)
≥ 65	1113 (1050–1176)	1184 (1056–1313)	288 (235–340)	1464 (1331–1597)	1578 (1397–1758)
≥ 75	1976 (1853–2100)	2107 (1855–2359)	536 (429–643)	2363 (2124–2603)	2935 (2573–3297)
65–74	321 (278–364)	359 (268–451)	75 (41–108)	534 (426–642)	364 (255–473)
75–84	1111 (1005–1217)	1212 (992–1432)	300 (208–391)	1556 (1323–1789)	1483 (1189–1777)
≥ 85	3994 (3666–4321)	4384 (3697–5071)	1177 (869–1486)	3889 (3356–4421)	6139 (5179–7099)
**Year 2: September 2023 to August 2024**					
≥ 25	539 (511–566)	697 (635–759)	129 (107–150)	667 (610–723)	754 (676–831)
≥ 65	1217 (1152–1281)	1507 (1366–1648)	292 (240–344)	1428 (1298–1557)	1767 (1578–1955)
≥ 75	2102 (1980–2224)	2628 (2363–2893)	506 (406–606)	2269 (2043–2495)	3136 (2771–3501)
65–74	357 (311–403)	426 (324–527)	95 (57–132)	507 (398–617)	489 (363–616)
75–84	1201 (1097–1305)	1707 (1462–1953)	319 (230–408)	1422 (1208–1637)	1482 (1201–1762)
≥ 85	4287 (3955–4620)	4943 (4250–5637)	1059 (764–1354)	3902 (3386–4417)	7117 (6078–8155)

Hospitalized RSV‐associated CAP and other ARI incidence rate per 100,000 person‐years (95% CI)					
Age group (years)	Overall	Asahikawa	Kamogawa	Kochi	Nagasaki
**Year 1: September 2022 to August 2023**					
≥ 25	12 (8–16)	0	5 (1–9)	20 (10–30)	13 (3–24)
≥ 65	29 (19–39)	0	13 (2–24)	49 (24–74)	23 (0–45)
≥ 75	63 (40–85)	0	28 (3–53)	100 (49–150)	49 (1–98)
65–74	0	0	0	0	0
75–84	30 (12–47)	0	15 (0–35)	57 (11–102)	0
≥ 85	150 (85–216)	0	65 (0–139)	183 (64–303)	171 (4–337)
**Year 2: September 2023 to August 2024**					
≥ 25	17 (12–21)	25 (13–38)	4 (0–8)	17 (8–26)	30 (14–46)
≥ 65	36 (25–48)	51 (24–77)	10 (0–19)	35 (14–56)	66 (29–103)
≥ 75	66 (44–88)	120 (61–178)	21 (0–41)	55 (19–92)	106 (37–175)
65–74	9 (2–17)	0	0	19 (0–41)	26 (0–56)
75–84	39 (20–58)	97 (37–157)	20 (0–42)	17 (0–42)	58 (1–114)
≥ 85	131 (70–191)	141 (18–265)	0	133 (35–232)	218 (27–409)

Hospitalized influenza‐associated CAP and other ARI incidence rate per 100,000 person‐years (95% CI)					
Age group (years)	Overall	Asahikawa	Kamogawa	Kochi	Nagasaki
**Year 1: September 2022 to August 2023**					
≥ 25	4 (2–7)	6 (0–12)	0	7 (1–13)	4 (0–10)
≥ 65	11 (4–17)	15 (0–30)	0	16 (2–31)	11 (0–27)
≥ 75	23 (9–37)	0	0	27 (1–53)	25 (0–59)
65–74	6 (0–12)	19 (0–40)	0	6 (0–17)	0
75–84	11 (0–21)	0	0	19 (0–45)	0
≥ 85	30 (1–60)	0	0	20 (0–60)	85 (0–203)
**Year 2: September 2023 to August 2024**					
≥ 25	31 (25–38)	25 (13–38)	0	45 (30–60)	49 (29–70)
≥ 65	71 (55–87)	65 (35–95)	0	99 (64–134)	104 (57–151)
≥ 75	153 (120–187)	90 (39–141)	0	160 (99–222)	165 (79–251)
65–74	24 (12–36)	32 (4–60)	0	32 (4–59)	44 (5–82)
75–84	56 (33–79)	0	0	96 (39–153)	101 (26–175)
≥ 85	276 (188–363)	395 (189–602)	0	285 (141–430)	306 (80–532)

Abbreviations: ARI, acute respiratory infection; CAP, community‐acquired pneumonia; CI, confidence interval; RSV, respiratory syncytial virus.

The incidence rates of RSV‐associated CAP and other ARI hospitalizations among adults aged ≥ 65 years were 29 (95% CI: 19–39) and 36 (95% CI: 25–48) per 100,000 person‐years. Further age stratification among adults aged ≥ 65 years revealed an increasing incidence with age, peaking in those aged ≥ 85 years: 150 (95% CI: 85–216) and 131 (95% CI: 70–191) per 100,000 person‐years in the first and second year, respectively.

The incidence rates of influenza‐associated CAP and other ARI hospitalizations increased notably from the first to second year. Among adults aged ≥ 65 years, the incidence rose from 11 (95% CI: 4–17) to 71 (95% CI: 55–87) per 100,000 person‐years. The highest rate was observed in those aged ≥ 85 years: 30 (95% CI: 1–60) and 276 (95% CI: 188–363) per 100,000 person‐years in the first and second year, respectively.

Geographically, the incidence rates for total, RSV‐ and influenza‐associated CAP and other ARI were generally comparable across Asahikawa, Kochi, and Nagasaki, but consistently lower in Kamogawa. In the sensitivity analysis excluding Kamogawa, the recalculated incidence rates among adults aged ≥ 65 years per 100,000 person‐years in the first year excluding Kamogawa were 34 (95% CI: 21–48) for RSV‐associated, and 15 (95% CI: 6–24) for influenza‐associated CAP and other ARI. In the second year, the corresponding rates were 46 (95% CI: 31–62), and 96 (95% CI: 74–119), respectively.

IRRs for hospitalization with total, RSV‐associated, and influenza‐associated CAP and other ARI among older versus younger adults are shown in Table 3. Across both study years, incidence rates were consistently higher among older adults aged (≥ 65 years) than among younger adults aged (25–64 years). This pattern was observed across all outcomes, with statistically significant differences (p< 0.001), except for influenza‐associated CAP and other ARI in the first study year, where no cases were detected in the younger group.

**TABLE 3:** Incidence rate ratio for hospitalization with total, RSV‐associated, and influenza‐associated CAP and other ARI among older versus younger adults in Japan, September 2022–August 2024.

	Incidence rate per 100,000 person‐years (95% CI)	Incident rate ratio (95% CI)	*p‐value*
**Hospitalized CAP and other ARI**			
**Year 1: September 2022 to August 2023**			
Younger adults (aged 25–64)	166 (136–195)	Reference	
Older adults (aged ≥ 65)	1113 (1050–1176)	6.7 (6.4–7.1)	< 0.001
**Year 2: September 2023 to August 2024**			
Younger adults (aged 25–64)	214 (180–247)	Reference	
Older adults (aged ≥ 65)	1217 (1152–1281)	5.7 (5.4–6.0)	< 0.001
**Hospitalized RSV‐associated CAP and other ARI**			
**Year 1: September 2022 to August 2023**			
Younger adults (aged 25–64)	3 (0–7)	Reference	
Older adults (aged ≥ 65)	29 (19–39)	10.4 (7.0–15.5)	< 0.001
**Year 2: September 2023 to August 2024**			
Younger adults (aged 25–64)	7 (1–13)	Reference	
Older adults (aged ≥ 65)	36 (25–48)	5.3 (4.0–6.9)	< 0.001
**Hospitalized influenza‐associated CAP and other ARI**			
**Year 1: September 2022 to August 2023**			
Younger adults (aged 25–64)	0	Reference	
Older adults (aged ≥ 65)	11 (4–17)	NA	NA
**Year 2: September 2023 to August 2024**			
Younger adults (aged 25–64)	12 (4–21)	Reference	
Older adults (aged ≥ 65)	71 (55–87)	5.7 (4.7–6.9)	< 0.001

Abbreviations: ARI, acute respiratory infection; CAP, community‐acquired pneumonia; CI, confidence interval; RSV, respiratory syncytial virus.

## 4 Discussion

To our knowledge, this is the first study to evaluate the incidence of RSV‐associated hospitalization as well as influenza‐associated hospitalizations among adults in Japan during and after the COVID‐19 pandemic.

The incidence of adult RSV‐associated hospitalizations has been estimated in several countries. However, direct comparisons are challenging due to differences in methodology, patient demographics, and hospitalization practices. Among studies using active surveillance during and after the COVID‐19 pandemic, our estimates were comparable to those reported from the UK [17], and lower than those from the US and Germany [19, 20]. A report from the UK estimated that the incidence of RSV‐associated hospitalizations among adults aged ≥ 65 years ranged from 33 to 53 per 100,000 person‐years from the 2017 to 2023 season, and 65 per 100,000 person‐years among adults aged ≥ 75 years during the 2022–2023 season [17], which was comparable to our study results, based on surveillance during the RSV season (week 40 to week 20 of the following year). In contrast, a US study estimated incidence rates of RSV‐associated hospitalizations at 98.5 and 160.9 per 100,000 for adults aged ≥ 65 years during the 2021–2022 and 2022–2023 seasons, respectively [19]. A German study reported 134.1 per 100,000 for adults aged ≥ 65 years from 2021 to 2023 [20]. Both the US and German studies adjusted for test sensitivity [8] and the potential underdetection associated with nasal swab sampling [20, 34].

While abundant epidemiological data on influenza, such as positivity percentage [35, 36] and disease burden [37, 38], are available, data on RSV, including RSV‐positivity percentage among hospitalized adults, remain limited in Japan both before and after the COVID‐19 pandemic. Two studies conducted in Japan during the postpandemic era reported RSV‐positivity percentages similar to our study [23, 24, 25], although none of them reported the incidence rate. One study reported a percentage of 2.1% among hospitalized patients aged ≥ 50 years with respiratory symptoms [24]; another study reported a percentage of 2.6% among outpatients aged ≥ 60 years with respiratory symptoms from December 2022 to November 2023 [23]. Positivity percentages reported prior to the COVID‐19 pandemic were slightly higher. The initial APSG‐J study conducted from 2011 to 2013 reported an RSV positivity of 5% among inpatients with respiratory symptoms [27]. Given that our results may still be influenced by the lingering effects of the COVID‐19 pandemic, continued surveillance is essential to accurately assess the burden of RSV in the adult population.

The long‐standing nationwide influenza vaccination program has been in place, whereas RSV vaccination has only recently become available in Japan, with very low uptake. Given these differences, the ability to compare the diseases' burden is limited; however, it is still useful for prioritizing preventive strategies. Some studies have reported higher morbidity and mortality in patients hospitalized with RSV compared with those hospitalized with influenza [39, 40]. In contrast, a recent systematic review found that the incidence rates of RSV‐associated hospitalizations and mortality were comparable to those of influenza [41]. In our study, the incidence rate of RSV‐associated hospitalizations was higher than that of influenza in the first year, whereas it was lower in the second year, particularly among older adults. Regarding clinical outcomes, although in‐hospital mortality was higher among RSV‐positive patients than among influenza‐positive patients, case numbers were small. Our study period overlapped with the implementation of strict public health measures, and observed results may have been influenced by changes related to the COVID‐19 pandemic. Our surveillance is ongoing and will continue through the third year of the study, which will provide more stable estimates of RSV‐ and influenza‐associated disease burden and help evaluate the potential impact of future RSV vaccine implementation in Japan.

Our study has several limitations. First, recruitment was limited to seven hospitals across four catchment areas, which may limit generalizability. Kamogawa area had lower incidence of hospitalizations with CAP and other ARI, possibly due to strict admission criteria at the hospital, and the rural and relatively isolated location. However, as noted in the Results, this had a limited impact on the overall incidence of RSV‐associated hospitalizations and our conclusions. Second, approximately half of the hospitalized CAP and other ARI patients did not undergo multiplex PCR. As incidence in this study was estimated based on etiologically confirmed cases, we did not apply statistical approaches such as multiple imputation. Nevertheless, the baseline characteristics of those tested and untested were largely compatible (Supplementary Section 2, Supplementary Table S1), supporting the generalizability of PCR findings. Third, our estimates of CAP and other ARI hospitalization incidence relied on available governmental data, which may have introduced some uncertainty. Fourth, the incidence of RSV‐associated hospitalizations may have been underestimated due to limited sensitivity of our diagnostic testing [19, 20, 34]. Fifth, our study included patients with ARI symptoms, possibly missing RSV‐associated hospitalizations without overt respiratory symptoms. Some studies estimating incidence included cardiopulmonary disease exacerbation, such as chronic obstructive pulmonary disease (COPD) and congestive heart failure (CHF), regardless of other symptoms [18, 42], while our study included such exacerbations only when accompanied by an ARI diagnosis, which may have underestimated the incidence of RSV‐associated hospitalizations. Sixth, our incidence estimation relied on government‐designated secondary medical areas as catchment areas and required assumptions regarding the representativeness of the proportion of CAP and other ARI hospitalizations among all hospitalizations across hospitals. Although we restricted the study population to residents within each hospital's catchment area and adjusted the total number of hospitalizations in each study hospital using government‐reported data on the proportion of hospitalizations from outside the catchment area at the secondary medical area level, we could not directly quantify the proportion of CAP and other ARI hospitalizations among all hospitalizations within each catchment area. In addition, heterogeneity in hospital functions may have contributed to the overestimation or underestimation of incidence.

In conclusion, we successfully evaluated the incidence of adult RSV‐associated CAP and other ARI hospitalizations in Japan using active surveillance. Our study provides essential evidence to inform discussions on the implementation of novel RSV vaccination policies for older adults.

## Author Contributions

**Haruka Maeda:** conceptualization, methodology, data curation, investigation, validation, formal analysis, writing – original draft. **Shingo Masuda:** investigation, data curation. **Bhim Gopal Dhoubhadel:** data curation, investigation. **Yuka Fujita:** investigation, data curation. **Yuji Akiba:** investigation, data curation. **Yutaka Nishigaki:** investigation, data curation. **Kei Nakashima:** investigation, data curation. **Hiroyuki Ito:** investigation, data curation. **Masayuki Nogi:** investigation, data curation. **Yoshihito Otsuka:** investigation, data curation. **Masayuki Ishida:** investigation, data curation. **Eiji Takeuchi:** investigation, data curation. **Norichika Asoh:** investigation, data curation. **Toyomitsu Sawai:** investigation, data curation. **Koichi Hayakawa:** investigation, data curation. **Eileen M. Dunne:** conceptualization, methodology, data curation, investigation, project administration. **Claudia Schwarz:** conceptualization, methodology, data curation, investigation, project administration. **Bradford D. Gessner:** methodology, investigation. **Elizabeth Begier:** methodology, investigation. **Shuhei Ito:** methodology, investigation. **Ataru Igarashi:** methodology, investigation. **Shinobu Osanai:** methodology, investigation. **Konosuke Morimoto:** conceptualization, methodology, data curation, investigation, project administration. **Koya Ariyoshi:** conceptualization, methodology, data curation, investigation, project administration. Other members of APSG‐J2: data curation, investigation.

## Funding

This work was supported as a research collaboration between Pfizer and Nagasaki University. Nagasaki University is the study sponsor. This work was supported by Pfizer (70109063).

## Ethics Statement

This study was approved by the Institutional Review Board of the Institute of Tropical Medicine, Nagasaki University with a centralized review (Approval No.: 220616247). With this approval, the investigators at the six sites obtained permission to conduct the study. In the remaining site, the study was approved by its individual review board following the approval by the centralized review (Approval No.: 22‐07‐F).

## Consent

Informed consent was obtained from a portion of the participants. For patients with whom consent could not be coordinated, enrollment followed an “opt‐out” approach in line with Japanese research ethics guidelines. For individuals who declined participation after being informed, minimal data (age, sex, and diagnosis) were collected to support incidence estimation, as approved by the Institutional Review Board of the Institute of Tropical Medicine, Nagasaki University with a centralized review (Approval No.: 220616247).

## Conflicts of Interest

E.M.D., C.S., B.D.G., E.B., and S.I. are Pfizer employees and may own Pfizer stock.

## Supporting information


Supplementary Section 1.
Supplementary Section 2.
**Table S1:** Characteristics of adults hospitalized with CAP and other ARI, with and without multiplex PCR, and those with only age, sex, and diagnosis information available, in Japan, September 2022–August 2024.
**Table S2:** Sample types for multiplex PCR from adult hospitalizations with CAP and other ARI in Japan, September 2022–August 2024.
**Table S3:** Positivity of RSV and influenza by sample types from adult hospitalizations with CAP and other ARI in Japan, September 2022–August 2024.
**Table S4:** Signs and symptoms of adults hospitalized with CAP and other ARI who underwent multiplex PCR, with RSV‐ and influenza‐positive CAP and other ARI in Japan, September 2022–August 2024.
**Table S5:** Classification of Nursing Care Level in Japan.
**Figure S1:** Study flow and surveillance arm.

## Data Availability

The data supporting the findings of this study are not available as consent for data sharing was not obtained.
