# Supplementary material for: Incidence of RSV‐ and Influenza‐Associated Hospitalizations With Community‐Acquired Pneumonia and Other Acute Respiratory Infection Among Adults in Japan in 2022–2024: APSG‐J2 Study
Source: Influenza Other Respir Viruses. 2026 Mar 15;20(3):e70238. doi: 10.1111/irv.70238 (PMC13098130; doi:10.1111/irv.70238)
Supplement: Supplementary file 1 — Supplementary Section 1. Supplementary Section 2. Table S1: Characteristics of adults hospitalized with CAP and other ARI, with and without multiplex PCR, and those with only age, sex, and diagnosis information available, in Japan, September 2022–August 2024. Table S2: Sample types for multiplex PCR from adult hospitalizations with CAP and other ARI in Japan, September 2022–August 2024. Table S3: Positivity of RSV and influenza by sample types from adult hospitalizations with CAP and other ARI in Japan, September 2022–August 2024. Table S4: Signs and symptoms of adults hospitalized with CAP and other ARI who underwent multiplex PCR, with RSV‐ and influenza‐positive CAP and other ARI in Japan, September 2022–August 2024. Table S5: Classification of Nursing Care Level in Japan. Figure S1: Study flow and surveillance arm. [file IRV-20-e70238-s001.docx]

**Supplementary Materials**

**Table of Contents**

[Supplementary Section 1. Study Eligibility Criteria, Catchment Area Definition, and Estimation of Hospitalization Denominators 2](#_Toc217055742)

[Supplementary Section 2. Characteristics of adults hospitalized with CAP and other ARI, with and without multiplex PCR, and those with only age, sex, and diagnosis information available, in Japan, September 2022–August 2024 4](#_Toc217055743)

[Table S1. Characteristics of adults hospitalized with CAP and other ARI, with and without multiplex PCR, and those with only age, sex, and diagnosis information available, in Japan, September 2022–August 2024 5](#_Toc217055744)

[Table S2. Sample types for multiplex PCR from adult hospitalizations with CAP and other ARI in Japan, September 2022–August 2024 7](#_Toc217055745)

[Table S3. Positivity of RSV and influenza by sample types from adult hospitalizations with CAP and other ARI in Japan, September 2022–August 2024 8](#_Toc217055746)

[Table S4. Signs and symptoms of adults hospitalized with CAP and other ARI who underwent multiplex PCR, with RSV- and influenza- positive CAP and other ARI in Japan, September 2022–August 2024 9](#_Toc217055747)

[Table S5. Classification of Nursing Care Level in Japan 10](#_Toc217055748)

[Figure S1. Study flow and surveillance arm 11](#_Toc217055749)

# Supplementary Section 1. Study Eligibility Criteria, Catchment Area Definition, and Estimation of Hospitalization Denominators

1. **Inclusion and exclusion criteria for APSG-J2**

The inclusion criteria were as follows: patients aged ≥18 years who met at least one of the following conditions: (1) clinical signs or symptoms of acute respiratory infection (ARI), (2) a diagnosis of pneumonia or ARI made by the attending physician, or (3) exacerbation of underlying cardiopulmonary disease with a concurrent diagnosis of ARI. Radiographic evaluation (chest X-ray or computed tomography [CT]) was also required. ARI signs or symptoms were defined as the presence of at least one of the following: fever (≥38°C), cough, sputum production, pleuritic chest pain, dyspnea, tachypnea (respiratory rate >20/min), hypoxemia (SpO₂ <93%), nasal congestion, rhinorrhea, sore throat, or hoarseness.

The exclusion criteria were as follows: a positive severe acute respiratory syndrome coronavirus 2 (SARS-CoV-2) test result at admission; pneumonia occurring more than 48 hours after hospital admission; residence outside the hospital’s publicly designated secondary medical area; prior enrollment in the study within the past 14 days; or a diagnosis of pulmonary tuberculosis or chronic pulmonary nontuberculous mycobacterial infection. At the Kamogawa site, patients from outside the catchment area were included to calculate the proportion of RSV-positive or influenza-positive CAP/ARI hospitalizations among CAP/ARI patients who underwent multiplex PCR, because a large number of patients resided outside the catchment area; however, these patients were excluded from incidence estimates.

1. **Definition of “Niji-Iryoken” (secondary medical area)**

In Japan, 344 Niji-Iryoken areas (secondary medical areas) were established in April 2013 as a framework for planning and coordinating the provision of medical care and related services in the country. Prefectural public health departments designate the required number of hospital beds using this area as the basic unit in accordance with the Japanese Medical Care Act (a national law). The number of outpatients and inpatients is made publicly available each year. The population size of each secondary medical area is determined based on the national census. In this study, secondary medical areas were used as catchment areas to define the denominator for incidence estimation.

1. **Estimation of the annual total number of hospitalizations in each catchment area (secondary medical area)**

The total number of hospitalizations in each catchment area was derived from the Patient Survey conducted by the Ministry of Health, Labour and Welfare between September 1 and 30, 2023 (1). As only September discharge data was available at the catchment area level, we used national discharge data covering all 12 months to derive monthly-to-September discharge ratios (2). These ratios were applied to the September discharge counts of each catchment area to estimate monthly discharges, assuming that local seasonal patterns followed national trends. The estimated monthly values were summed to obtain annual hospitalization counts for each catchment area. To restrict the analysis to general medical beds, we excluded discharges from psychiatric, long-term care, and tuberculosis-designated beds by adjusting the total number using the respective proportions.

References:

1. Ministry of Health, Lbour and Welfare. Patient Survey, 2023. Available from: https://www.e-stat.go.jp/stat-search/files?page=1&query=%E6%8E%A8%E8%A8%88%E9%80%80%E9%99%A2%E6%82%A3%E8%80%85%E6%95%B0%E3%80%80%E4%BA%8C%E6%AC%A1%E5%8C%BB%E7%99%82%E5%9C%8F&layout=dataset&metadata=1&data=1

2. Statistics Bureau of Japan. 2020 Population Census of Japan. Available from: https://www.e-stat.go.jp/stat-search/files?page=1&layout=datalist&toukei=00200521&tstat=000001136464&cycle=0&year=20200&month=24101210&tclass1=000001154387&tclass2=000001159626&tclass3val=0.

# Supplementary Section 2. Characteristics of adults hospitalized with CAP and other ARI, with and without multiplex PCR, and those with only age, sex, and diagnosis information available, in Japan, September 2022–August 2024

Supplementary Table 1 shows the characteristics of adults hospitalized with community-acquired pneumonia (CAP) and other acute respiratory infection (ARI), comparing those who underwent multiplex polymerase chain reaction (PCR) with those who did not. Overall, patients who received multiplex PCR were generally similar to those who were not tested. However, untested patients were slightly older (aged ≥65 years: 90.6% vs. 87.7%), more likely to be female (40.5% vs. 35.9%), more likely to reside in a nursing home (30.0% vs. 17.5%), and less likely to have one or more underlying medical conditions (77.2% vs. 82.3%) (Supplementary Table 1).

# Table S1. Characteristics of adults hospitalized with CAP and other ARI, with and without multiplex PCR, and those with only age, sex, and diagnosis information available, in Japan, September 2022–August 2024

|  | **CAP and other ARI hospitalizations with multiplex PCR, no. (%)** | **CAP and other ARI hospitalizations without multiplex PCR, no. (%)** | **CAP and other ARI hospitalizations of patients in the Declined Consent Arm^a^, no. (%)** |
| --- | --- | --- | --- |
| Overall | 1,499 (100) | 1,369 (100) | 179 (100) |
| Median age (IQR), years | 81 (73–88) | 82 (75–89) | 80 (74–86) |
| Age group in years |  |  |  |
| 18–64 | 184 (12.3) | 129 (9.4) | 17 (9.5) |
| $\geq$65 | 1,315 (87.7) | 1,240 (90.6) | 162 (90.5) |
| Sex |  |  |  |
| Men | 961 (64.1) | 815 (59.5) | 122 (68.2) |
| Women | 538 (35.9) | 554 (40.5) | 57 (31.8) |
| Underlying medical conditions^b^ |  |  |  |
| Any | 1,233 (82.3) | 1,057 (77.2) | N/A |
| Asplenia | 1 (0.1) | 0 | N/A |
| Cancer | 234 (15.6) | 245 (17.9) | N/A |
| Leukemia | 2 (0.1) | 3 (0.2) | N/A |
| Lymphoma | 10 (0.7) | 8 (0.6) | N/A |
| Multiple myeloma | 1 (0.1) | 6 (0.4) | N/A |
| Immunosuppressive drug use | 73 (4.9) | 69 (5.0) | N/A |
| Organ transplantation | 2 (0.1) | 0 | N/A |
| Diabetes mellitus | 348 (23.2) | 317 (23.2) | N/A |
| Nephrotic syndrome | 3 (0.2) | 5 (0.4) | N/A |
| Chronic heart failure | 264 (17.6) | 266 (19.4) | N/A |
| Chronic heart disease other than chronic heart failure | 343 (22.9) | 284 (20.7) | N/A |
| Chronic lung disease (including asthma) | 604 (40.3) | 353 (25.8) | N/A |
| Chronic renal failure | 146 (9.7) | 114 (8.3) | N/A |
| Chronic live disease | 68 (4.5) | 55 (4.0) | N/A |
| Obesity (BMI ≥40) | 4 (0.3) | 1 (0.1) | N/A |
| Cerebrospinal fluid leakage | 1 (0.1) | 1 (0.1) | N/A |
| Cerebrovascular disease | 270 (18.0) | 262 (19.1) | N/A |
| Current smokers |  |  |  |
| Yes | 134 (8.9) | 145 (10.6) | N/A |
| No | 1,284 (85.7) | 979 (71.5) | N/A |
| Unknown | 81 (5.4) | 245 (17.9) | N/A |
| Nursing-home residents | 262 (17.5) | 411 (30.0) | N/A |
| Nursing care level^c^ |  |  |  |
| Independent | 804 (53.6) | 598 (43.7) | N/A |
| Support (1–2) | 145 (9.7) | 99 (7.2) | N/A |
| Care (1–5) | 548 (36.5) | 654 (47.8) | N/A |
| Unknown | 2 (0.1) | 18 (1.3) |  |
| Prior hospitalization within the past 90 days |  |  |  |
| Yes | 290 (19.3) | 258 (18.8) | N/A |
| No | 1,185 (79.1) | 1,059 (77.2) | N/A |
| Unknown | 24 (1.6) | 52 (3.8) | N/A |
| Preceding antibiotics use within 14 days |  |  |  |
| Yes | 298 (19.9) | 229 (16.7) | N/A |
| No | 1,188 (79.3) | 1,102 (80.5) | N/A |
| Unknown | 13 (0.9) | 38 (2.8) | N/A |
| Study period |  |  |  |
| Year 1 (September 2022, to August 2023) | 754 (50.3) | 595 (43.5) | N/A |
| Year 2 (September 2023, to August 2024) | 745 (49.7) | 774 (56.5) | N/A |
| Area |  |  | N/A |
| Asahikawa | 218 (14.5) | 572 (41.8) | N/A |
| Kamogawa | 198 (13.2) | 274 (20.0) | N/A |
| Kochi | 780 (52.0) | 182 (13.3) | N/A |
| Nagasaki | 303 (20.2) | 341 (24.9) | N/A |
| Diagnosis |  |  |  |
| Community-acquired pneumonia | 1,359 (90.7) | 1,237 (90.4) | 152 (84.9) |
| Other acute respiratory infection | 140 (9.3) | 132 (9.6) | 27 (15.1) |

Abbreviations: CAP, community-acquired pneumonia; ARI, acute respiratory infection; PCR, polymerase chain reaction; IQR, interquartile range; BMI, body mass index.

a: For patients who declined to participate were included in the Declined Consent Arm, only age, sex, and diagnosis information of them were registered (See Supplementary Figure 1).

b: No patients had sickle cell disease, HIV infection, Hodgkin’s disease, or cochlear implant.

c: Nursing care level was determined based on the Long-Term Care Insurance System by the Ministry of Health, Labour and Welfare. Details are provided in Supplementary Table 5.

# Table S2. Sample types for multiplex PCR from adult hospitalizations with CAP and other ARI in Japan, September 2022–August 2024

| **Sample type, no.** | **Overall, no. (%)** | **Sep 2022–Aug 2023,**  **no. (%)** | **Sep 2023–Aug 2024,**  **no. (%)** |
| --- | --- | --- | --- |
| Any | 1,499 | 754 | 745 |
| Sputum | 1,325 (88.4) | 698 (92.6) | 627 (84.2) |
| Saliva | 380 (25.4) | 89 (11.8) | 291 (39.1) |
| Nasopharyngeal swab | 108 (7.2) | 23 (3.1) | 85 (11.4) |

Abbreviations: PCR, polymerase chain reaction; CAP, community-acquired pneumonia; ARI, acute respiratory infection.

# Table S3. Positivity of RSV and influenza by sample types from adult hospitalizations with CAP and other ARI in Japan, September 2022–August 2024

| **Sample type** | **Period** | **No. tested** | **RSV-positive,**  **no. (%)** | **Influenza-positive, no. (%)** |
| --- | --- | --- | --- | --- |
| **Any** | Overall | 1,499 | 42 (2.8) | 49 (3.3) |
|  | Sep 2022**–**Aug 2023 | 754 | 17 (2.3) | 6 (0.8) |
|  | Sep 2023**–**Aug 2024 | 745 | 25 (3.4) | 43 (5.8) |
| **Sputum** | Overall | 1,325 | 39 (2.9) | 41 (3.1) |
|  | Sep 2022**–**Aug 2023 | 698 | 17 (2.4) | 5 (0.7) |
|  | Sep 2023**–**Aug 2024 | 627 | 22 (3.5) | 36 (5.7) |
| **Saliva** | Overall | 380 | 9 (2.4) | 18 (4.7) |
|  | Sep 2022**–**Aug 2023 | 89 | 1 (1.1) | 0 |
|  | Sep 2023**–**Aug 2024 | 291 | 8 (2.7) | 18 (6.2) |
| **Nasopharyngeal swab** | Overall | 108 | 4 (3.7) | 4 (3.7) |
|  | Sep 2022**–**Aug 2023 | 23 | 0 | 1 (4.3) |
|  | Sep 2023**–**Aug 2024 | 85 | 4 (4.7) | 3 (3.5) |

Abbreviations: RSV, respiratory syncytial virus; CAP, community-acquired pneumonia; ARI, acute respiratory infection.

# Table S4. Signs and symptoms of adults hospitalized with CAP and other ARI who underwent multiplex PCR, with RSV- and influenza- positive CAP and other ARI in Japan, September 2022–August 2024

|  | **Overall, no. (%)**  **(n=1,499)** | **RSV-positive, no. (%)**  **(n=42)** | **Influenza-positive, no. (%) (n=49)** |
| --- | --- | --- | --- |
| Fever (≥38℃) | 687 (45.8) | 28 (66.7) | 32 (65.3) |
| Cough | 620 (41.4) | 28 (66.7) | 34 (69.4) |
| Sputum | 556 (37.1) | 24 (57.1) | 27 (55.1) |
| Pleuritic chest paint | 84 (5.6) | 2 (4.8) | 3 (6.1) |
| Dyspnea | 672 (44.8) | 17 (40.5) | 30 (61.2) |
| Tachypnea (respiratory rate >20/min) | 174 (11.6) | 7 (16.7) | 8 (16.3) |
| Nasal congestion | 11 (0.7) | 1 (2.4) | 2 (4.1) |
| Rhinorrhea | 70 (4.7) | 5 (11.9) | 4 (8.2) |
| Sore throat | 71 (4.7) | 3 (7.1) | 5 (10.2) |
| Hoarseness | 10 (0.7) | 1 (2.4) | 0 |

Abbreviations: CAP, community-acquired pneumonia; ARI, acute respiratory infection; PCR, polymerase chain reaction; RSV, respiratory syncytial virus

# Table S5. Classification of Nursing Care Level in Japan

| **Care level** |  |
| --- | --- |
| Independent | Capable of performing daily activities without assistance. |
| Support 1 | Capable of performing daily activities independently but requires occasional assistance. |
| Support 2 | Requires some assistance with daily activities, with a high potential for improvement without the need for nursing care. |
| Nursing Care 1 | Experiences unsteadiness when standing or walking.  Requires partial assistance with daily activities such as toileting and bathing. |
| Nursing Care 2 | Has difficulty standing or walking independently.  Requires partial to full assistance with daily activities such as toileting and bathing. |
| Nursing Care 3 | Unable to stand or walk.  Requires full assistance with daily activities including toileting, bathing, and dressing/undressing. |
| Nursing Care 4 | Requires full assistance in all aspects of daily life, including toileting, bathing, and dressing/undressing. |
| Nursing Care 5 | Requires full assistance in all aspects of daily life and has significant difficulty with communication. |

This classification is defined by the Ministry of Health, Labour and Welfare in Japan.

# Figure S1. Study flow and surveillance arm


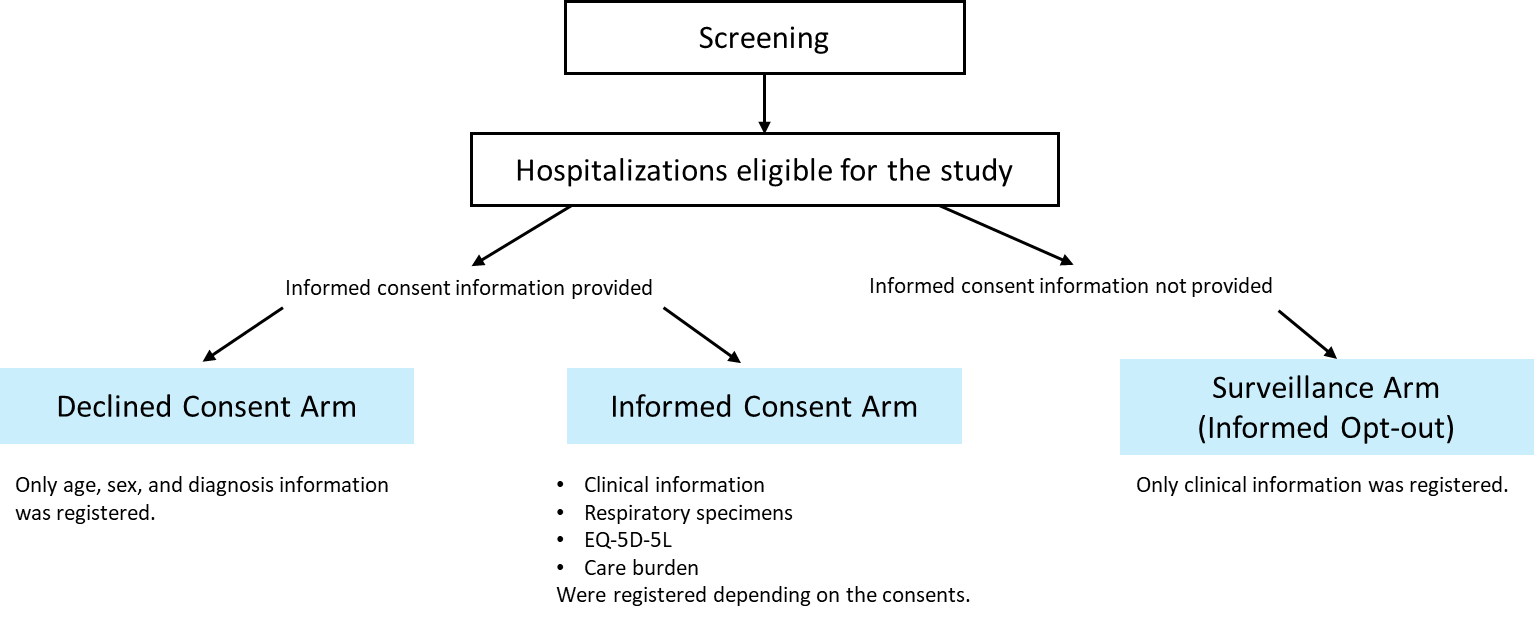


Following screening, hospitalizations eligible for the study were categorized into three groups based on the informed consent procedures. Patients who explicitly declined participation were assigned to the Declined Consent Arm. Those who provided informed consent were included in the Informed Consent Arm. Patients who were not directly approached for consent, in accordance with the Japanese guidelines on informed opt-out, were assigned to the Surveillance Arm (Informed Opt-out).
